# The efficacy of physiotherapy approaches in chronic tension-type headache: a systematic review and meta-analysis

**DOI:** 10.22514/jofph.2025.003

**Published:** 2025-03-12

**Authors:** Dilara Onan, Halime Arıkan, Esme Ekizoğlu, Bahar Taşdelen, Aynur Özge, Paolo Martelletti

**Affiliations:** ^1^Department of Physiotherapy and Rehabilitation, Faculty of Health Sciences, Yozgat Bozok University, 66100 Yozgat, Türkiye; ^2^Department of Physiotherapy and Rehabilitation, Faculty of Health Sciences, Tokat Gaziosmanpasa University, 60000 Tokat, Türkiye; ^3^Department of Neurology, Faculty of Medicine, Istanbul University, 34093 Istanbul, Türkiye; ^4^Department of Biostatistics and Medical Informatics, Faculty of Medicine, Mersin University, 33343 Mersin, Türkiye; ^5^Department of Neurology, Algology and Clinical Neurophysiology, Faculty of Medicine, Mersin University, 33343 Mersin, Türkiye; ^6^School of Health, Unitelma Sapienza University of Rome, 00161 Rome, Italy

**Keywords:** Chronic tension-type headache, Physiotherapy, Acupuncture, Exercise, Muscle relaxation, Meta-analysis, GRADE

## Abstract

**Background**: Although pharmacologic therapies are considered the first choice for the treatment of chronic tension-type headache (CTTH), physiotherapy and rehabilitation approaches are also used in the management of patients with CTTH. This study aimed to investigate the efficacy of physiotherapy approaches in CTTH through a systematic review and meta-analysis. **Methods**: The following electronic databases were searched, PubMed and Web of Science databases. Common primary outcomes from randomized controlled trials (RCTs) were changes in the intensity and duration of headaches, headache frequency, disability and headache impact. The methodologic quality (completeness of reporting and risk of bias) of trial reports included in systematic reviews was assessed using the Physiotherapy Evidence Database scale ratings. We also performed data synthesis and quantitative analysis of the eligible data. **Results**: Nine RCTs were included in the review. Seven studies related to intensity of headache (IH), three on headache frequency (HF), three on headache duration (HD), and two on headache impact were eligible for quantitative analysis. Analysis of the data showed that neck-shoulder strength exercises, electroacupuncture, and approaches targeting muscle relaxation improved the IH (−1.17 (−1.86, −0.49) *p * < 0.01) and reduced the HD (−0.71 (−1.31, −0.12), *p* = 0.02); the approaches targeting muscle relaxation and neck-shoulder strength exercises induced a significant decrease in the HF (−1.36 (−2.47, −0.26), *p* = 0.02) in patients with CTTH in comparison with the control groups. **Conclusions**: Neck-shoulder strength exercises and muscle relaxation are effective in reducing the intensity, duration, and frequency of headaches and electroacupuncture causes significant improvement in the duration and intensity of headaches in patients with CTTH. **The PROSPERO Registration**: PROSPERO number is CRD42023457085.

## 1. Introduction

Tension-type headache (TTH) is characterized by mild-to-moderate pain presenting in episodic and chronic forms. According to the International Classification of Headache Disorders (ICHD 3), chronic tension-type headache (CTTH) is defined as a headache occurring 15 days or more per month for more than 3 months, lasting hours to days or even being unremitting in some cases. Pain has characteristics similar to episodic forms [1, 2].

CTTH is believed to be caused by muscle tension in the head, neck or face. However, the exact mechanisms of CTTH are unknown [2] and may be multifactorial based on genetic predominance along with psychological cofounders, and hyperexcitability of peripheral and central pain pathways [3, 4]. Tenderness in the pericranial muscles is frequently observed in patients with TTH during acute headache episodes and sometimes even outside these periods, suggesting a possible involvement of myofascial tissues in the pathophysiology of TTH [5, 6]. Both the pericranial muscles and tendon insertions exhibit higher tenderness scores in patients with episodic TTH and CTTH compared with control subjects [7]. Patients with TTH exhibit reduced thickness in the longus colli and cervical multifidus muscles, as well as a lower pressure pain threshold, compared to healthy controls [8]. It remains under discussion whether muscle tenderness results from the pain or plays a role in triggering TTH attacks [9].

Myofascial trigger points, which are highly sensitive areas linked to a tight band (a region of muscle fibers with increased tension) in skeletal muscles that cause local and referred pain upon pressure, might be significant in the pathophysiology of TTH [10]. It is recommended that cervical musculoskeletal dysfunctions and the sensitivity that comes with them be resolved with non-pharmacologic treatment methods that include the spine in patients with CTTH [11, 12, 13]. When examining years lived with disability (YLD), TTH has an age-standardized YLD of 73.9/100,000 according to the last study in 2019 [14]. Thus, the diversity and complexity of the pathophysiologic aspects of CTTH, its burden on society, and the loss of workforce necessitate the use of multimodal therapeutic strategies to manage this disorder [15, 16].

In chronic headaches, which also include TTHs, the 2030 Agenda for Sustainable Development states that a series of method items should be addressed, including reducing the excessive use of acute drugs, and a multifaceted disease method that includes pharmacologic and non-pharmacologic treatment methods [17]. Although pharmacologic approaches are applied in the first step of the management of CTTH, non-pharmacologic approaches are also useful in maintaining the effectiveness and well-being of the treatment [16]. These non-pharmacologic therapies include theraupeutic patient education, behavioral treatments, physiotherapy approaches such as acupuncture, nerve electric stimulations, manual therapy, progressive muscle relaxation, and physical exercises [2, 15, 18, 19, 20, 21, 22, 23]. Considering the chronic state of pain, it creates a global burden and the incidence, prevalence, and disability of TTHs increase in adolescents and young adults, revealing the need to investigate effective intervention methods to reduce headache symptoms and lifelong effects [24, 25, 26, 27].

However, no systematic review and meta-analysis specifically addressing the effect of physiotherapy treatment on symptom improvement in CTTH was identified. Considering the evidence gap in the literature, our hypotheses were as follows: H0—According to the systematic review and meta-analysis review, physiotherapy approaches would not be effective on headache symptoms on CTTH; H1—According to the systematic review and meta-analysis review, physiotherapy approaches would be effective on headache symptoms on CTTH. With this information, the aim of this study was to investigate the efficacy of physiotherapy approaches on headache symptoms in CTTH through a systematic review and meta-analysis.

## 2. Materials and methods

We performed a systematic review of the relevant articles using the Preferred Reporting Items for Systematic Reviews and Meta-Analyses (PRISMA) guidelines (**Supplementary Table 1**) [28]. The PRISMA flow diagram is presented in Fig. 1. The study protocol was registered in the PROSPERO system (number: CRD42023457085, https://www.crd.york.ac.uk/prospero/).

**Fig. 1.** PRISMA flow diagram.

### 2.1 Review question

The review question was created using the PICOS (Participants, Intervention, Comparison, Outcome, Study design) process: Do physiotherapy approaches improve the intensity of headache, duration, disability, frequency, and quality of life (QoL) in patients with CTTH? (P: Patients with CTTH; I: Physiotherapy approaches; C: Comparison group (healthy or placebo or sham); O: Intensity of headache, duration, disability, frequency, QoL; S: This study included randomized controlled studies (RCTs) that compare the effectiveness of physiotherapy interventions on the intensity of headache, duration, disability, frequency, and QoL in CTTH).

### 2.2 Search strategy

The search was performed using the PubMed and Web of Science databases by two independent researchers with Ph.D. degrees, from the date the databases became available to 30 June 2024. After reviewing the articles included in the study, a meta-analysis was conducted using the available quantitative data. Keywords of the search strategy are given in the **Supplementary material**.

### 2.3 Eligibility criteria and study selection

The inclusion criteria were as follows: (1) Articles published CTTH in human RCTs in a peer-reviewed scientific journal; (2) Sufficient information to complete an analysis; (3) Use of any physiotherapy approach as an experimental treatment in individuals with CTTH; (4) Published in English; (5) Only adult participants (age 18 years and older); (6) All RCTs up to the last search date.

The exclusion criteria were as follows: (1) Articles published as animal studies, letters to the editor, abstracts, review articles, retrospective-prospective cohort studies, case reports/series/case-control studies, editorials, expert opinions, or instructional articles; (2) Age under 18 years; studies investigating patients with headache disorders other than CTTH; (3) Use of physiotherapy approaches for headaches other than CTTH or included treatments other than physiotherapy approaches.

In the first step of the study selection process, two independent researchers (both physiotherapists, DO and HA) with Ph.D. degrees screened the articles by reviewing the titles and abstracts according to the inclusion and exclusion criteria. In the second step, they examined the full texts of the remaining articles and determined whether they were suitable for the study. The reviewers resolved any discrepancies in ratings through verbal discussion. A consensus was reached for all studies during the initial meeting. There were no missing data or issues with the standardization of outcome units.

### 2.4 Methodologic quality assessment

The methodologic quality of the RCTs was also evaluated by the same independent researchers (DO and HA) using the Physiotherapy Evidence Database (PEDro scale) (https://pedro.org.au/), in the same manner that was described in our previous research [29]. The PEDro scale is an 11-item scale where each item has a YES = positive or NO = negative rating response. The total score shows the level; scores below 4 = poor, 4–5 = fair, 6–8 = good and 9–11 = are excellent quality [30].

### 2.5 Assessment of risk of bias

Risk of bias (RoB) assessment was performed by two independent authors (DO and HA) using the Cochrane RoB 2 tool [31]. In cases of disagreement, a third author (EE) was consulted to reach a decision. Five domains were examined: randomization process, deviations from the intended intervention (intention to treat), missing outcome data, measurement of outcome, and selection of reported outcomes. Studies were assessed as low risk, some concerns or high risk (Fig. 2).

**Fig. 2.** Risk of bias summary. D1a: Randomization process; D1b: Timing of identification or recruitment of participants; D2: Deviations from the intended interventions; D3: Missing outcome data; D4: Measurement of the outcome; D5: Selection of the reported result.

### 2.6 Data extraction

We performed data extraction from the RCTs to retrieve relevant information on the clinical findings and the trial characteristics. The country where the study was conducted, sample sizes, sex distribution, and the mean age of the participants, outcome measures, types and durations of interventions applied in each arm were all noted. Outcome measures of interest were changes in the frequency, duration, and intensity of headache attacks, and the impact of CTTH on the quality of life of the patients. Three researchers independently collected the necessary information from the included studies to ensure the consistency and accuracy of the extracted data. The Excel program was used in scanning, data extraction, and quality rating processes.

### 2.7 Level of evidence

The level of evidence for each outcome was evaluated using the Grading of Recommendations, Assessment, Development and Evaluations (GRADE) system. Five items were considered in determining the level of evidence: risk of bias, inconsistency, imprecision, indirectness and publication bias [32].

### 2.8 Data synthesis and analysis

The data for the quantitative assessment were extracted for primary outcomes. First, pre and post-mean values and standard deviations for experiment and control groups were extracted from the included RCTs, and then mean decreases from baseline were calculated. To impute standard deviations of the change from baseline, correlation coefficients between pre and post-measurements were required.

There was no prior knowledge to calculate the correlation coefficient between pre-post measurements, medium correlation coefficients (0, 50) were assumed, and then, standard deviations of the change from baseline for the experimental therapy and comparator groups were estimated as follows:

$$S{\hat{D}}_{E,change}=\sqrt{SD_{E,baseline}^{2}+SD_{E,after}^{2}-(2\times Corr_{E}\times SD_{E,baseline}\times SD_{E,after})}$$

$$S{\hat{D}}_{C,change}=\sqrt{SD_{C,baseline}^{2}+SD_{C,after}^{2}-(2\times Corr_{C}\times SD_{C,baseline}\times SD_{C,after})}$$

The level of heterogeneity in the studies was assessed using the *I*^2^ statistic. Heterogeneity classification is: 0–40% = not important; 30–60% = moderate; 50–90% = substantial, 75–100% = considerable [33]. The findings from the included studies were comprehensively evaluated through data analysis and synthesis. If there was at least substantial heterogeneity, an overall effect evaluation was performed according to the random effects model. A funnel plot graph was used to determine publication bias [34]. Analysis of the data was performed using the meta package of R (v4.17-0) [35].

## 3. Results

A total of 237 articles were screened from two different database tools according to the inclusion criteria. Nine RCTs were eligible for narrative review. All article selection stages of the study are presented in the PRISMA flow diagram (Fig. 1).

The included studies are summarized in Table 1 (Ref. [2, 36, 37, 38, 39, 40, 41, 42, 43]) in terms of country of origin, number of participants in each arm, mean age, sex distribution, management in intervention and control groups, duration of treatment, and outcome measures. All but one study [36] (that did not give any data on sex distribution) included mostly female participants and total sample sizes ranged from 25 to 169 individuals. The duration of the treatment ranged between 3 and 12 weeks, with a maximum follow-up period (FUP) of 4–18 weeks except in two studies where the FUP duration was not reported [37, 38]. Outcome measures varied among these studies. Three reported duration of headache in hours [36, 38, 39], six studies reported headache frequency as monthly headache days [2, 38, 40], weekly headache-free days [37], weekly headaches [39], and headache attacks over 2 weeks [36], nine studies assessed intensity of headache using visual analog scales (VAS) or numerical pain rating scales (NPRS) [36, 38, 39, 41, 42, 43], a numerical pain index [40], point rating scale (ranging between 0 and 10) [37], the Wong-Baker Faces Pain Scale (WBFPS) (ranging between 0–10) [2], four studies assessed the headache impact using the Headache Impact Test-6 (HIT-6) [2, 38, 42, 43], and another one evaluated disability using the Headache Disability Index (HDI) [43]. We reviewed the common outcome measures reported in these studies and performed further quantitative analyses accordingly.

**Table 1.** Randomized clinical trials of physiotherapy approaches in chronic tension type headache: characteristics of trials.

Authors	Country	Sample size, n	Age, mean ± SD; median (range)	% Female	Intervention Group	Control Group	Tx Duration	Max FUP	Outcome Measures
Berggreen *et al*. [41] 2012	Denmark	I: 20^a^ C: 19^b^	I: 38.8 ± 13.7; C: 42.3 ± 10.2	I: 100% C: 100%	Trigger point massage	No treatment	10 w	18 w	Headache intensity (VAS); Change in number of myofascial trigger points; Use of medication (mg/day); Quality of Life (SF-36)
Chassot *et al*. [42] 2015*	Brazil	I: 17 C: 17	I: 39.11 ± 10.5 C: 41.44 ± 10.5	I: 100% C: 100%	Electroacupuncture	Sham	5 w	12 w	Headache intensity (VAS); Pain assessment (pain diaries, BDNF); Headache impact (HIT-6)
Gopichandran *et al*. [2] 2021*	India	I: 84 C: 85	I: 44.21 ± 10.7 C: 46.16 ± 11.12	I: 53.5% C: 60%	Muscle relaxation and deep breathing exercise	No intervention	12 w	12 w	Headache intensity (WBFPS); Headache frequency (headache days/month); Headache impact (HIT-6); Sleep (PSQI)
Kiran *et al*. [39] 2014*	India	I: 30 C: 20	32.06 ± 9.43 (all participants)	N.A.	Raj yoga and routine medical treatment (analgesics and muscle relaxants)	Routine medical treatment (analgesics and muscle relaxants)	8 w	8 w	Headache intensity (VAS); Headache frequency (headaches/week); Headache duration (hours); Headache index (multiplying intensity of headache and frequency of headache per week)
Pérez-Llanes *et al*. [43] 2022*	Spain	I: 13 C: 12	I: 43.3 ± 18.07 C: 46.2 ± 16.37	I: 76.9% C: 91.6%	Suboccipital muscle inhibition combined with interferential current + Pharmacologic Tx	Pharmacologic Tx	4 w	4 w	Headache intensity (VAS); Disability (HDI); Headache impact (HIT-6)
Mohamadi *et al*. [40] 2020*	Iran	I: 13 C: 13	I: 39 ± 11; C: 38 ± 9	I: 84.6% C: 76.9%	Positional Release Technique^†^ and Ibuprofen (200 mg) (if needed)	Ibuprofen (200 mg) (if needed)	5 w	5 w	Headache intensity (Numerical pain index); Headache frequency (headache days/month); Pain assessment (MPQ) Changes in metabolite profile; Local and distal pressure pain thresholds
Rokicki *et al*. [37] 1997	USA	I: 30 C: 14	I: 19 ± N.A.; C: 18.64 ± N.A.	I: 86% C: 86%	Relaxation/EMG Biofeedback	No intervention	3 w	NR	Headache intensity (11-point rating scale, 0–10 ranges); Headache frequency (headache-free days/week); Headache activity score Use of medication; EMG activity; Cognitive changes (Headache-Specific Locus of Control Scale); Individual’s beliefs (Headache Self-Efficacy Scale)
Wang *et al*. [36] 2007*	Denmark	I: 18 C: 18	I: Women: 38.3 ± 4.7; Men: 47.2 ± 4.7; C: Women: 54.9 ± 3.9; Men: 51.5 ± 7.4	I: 44% C: 55%	Acupuncture-like electrical stimulation	Sham stimulation	4 w	12 w	Headache intensity (VAS); Headache frequency (headache attacks over 2 w); Daily headache duration (hours/day); Use of medication (number of pills per 2 w)
Martín-Vera *et al*. [38] 2023*	Spain	I: 20 C: 20	I: 33.9 ± 12.2; C: 40.1 ± 14	I: 85% C: 75%	Neck-shoulder strength exercises	Daily activities without monitoring	12 w	NR	Headache intensity (VAS); Headache frequency (days/month); Headache duration (hours); Cervical muscle thickness (cm); Cervical range of motion (degrees); Pain pressure threshold (kg/cm^2^)

BDNF: Brain-derived neurotrophic factor; C: Control group; EMG: electromyography; FUP: follow-up period; HDI: Headache Disability Inventory; HIT-6: Headache Impact Test-6; SF-36: Short Form 36; I: Intervention group; mg: milligram; MPQ: McGill’s Pain Questionnaire; PSQI: Pittsburgh Sleep Quality Index; SD: standard deviation; VAS: Visual Analog Scale (0–10/0–100); Tx: Treatment; w: weeks; WBFPS: Wong-Baker Faces Pain Scale (0–10); NR: Not reported; N.A.: Not Applicable. *Trials included in the quantitative analyses. ^a^: Drop-out in the intervention group; ^b^: Drop-out in the control group during the study period. ^†^Applied on the trapezius, sternocleidomastoid, obliquscapitis superior, rectus capitis anterior, rectus capitis posterior, interspinalis and multifidus muscles.

### 3.1 Description of the studies

The following physiotherapy approaches were performed to treat patients with CTTH in the intervention arms: Acupuncture-like electrical stimulation [36], trigger point massage [41], suboccipital muscle inhibition combined with interferential current + pharmacologic treatment [43], electroacupuncture [42], positional release technique and ibuprofen [40], relaxation/electromyography (EMG) biofeedback [43], Raj yoga and routine and medical treatment [39], muscle relaxation and deep breathing exercise [2], and neck-shoulder strength exercises [38] (Fig. 3).

**Fig. 3.** Summarized physiotherapy intervention approaches in CTTH. EMG: electromyography. The images were specifically created using BioRender (https://www.biorender.com/).

Wang *et al*. [36] investigated the efficacy of acupuncture-like electrical stimulation on CTTH. The study comprised two arms that received acupuncture-like electrical stimulation and sham stimulation, for 4 weeks. Headache frequency, intensity, and duration of headache decreased at the endpoint in both arms (*p * < 0.05 for both). Although the improvements were greater in the intervention group, the differences in the outcomes did not reach statistical significance. The authors concluded that the acupuncture-like electrical stimulation might be effective in CTTH [36]. Another study used also electroacupuncture to treat patients with CTTH for 5 weeks and compared its effect to that of sham stimulation. Real electroacupuncture was more effective than sham acupuncture in reducing headache intensity (as assessed using VAS) (*p* = 0.005). One of the outcomes was cited as headache impact assessment using HIT-6. However, the authors did not report the post-treatment data [42].

Trigger point massage was applied to patients with CTTH in the study of Berggreen* et al.* [41] over a period of 10 weeks. There was an intervention group (trigger point massage) and a control group (no treatment). The intervention group had a more significant improvement in morning VAS scores showing headache intensity compared with the control group, which did not receive any treatment for their headaches (*p* = 0.047). The SF-36 scores, assessing the QoL, did not vary between the two arms.

In the study by Pérez-Llanes *et al*. [43] suboccipital muscle inhibition combined with interferential current and pharmacologic treatment was used for 4 weeks in the intervention group, and the control group received only pharmacologic treatment. Endpoint data showed no significant improvement in headache intensity (as assessed using VAS) (*p* = 0.18) between the groups. However, there were improvements in disability as assessed using the Headache Disability Index (HDI) and Neck Disability Index (NDI) scores (*p* = 0.022 and *p* = 0.001, respectively) and headache impact as assessed using HIT-6 scores (*p* = 0.037). These results favored the beneficial effect of the combination of interferential current and pharmacologic treatment to reduce disability and improve the headache impact in CTTH.

In the study by Pérez-Llanes *et al*. [43] suboccipital muscle inhibition combined with interferential current and pharmacologic treatment was used for 4 weeks in the intervention group, and the control group received only pharmacologic treatment. Endpoint data showed no significant improvement in headache intensity (as assessed using VAS) (*p* = 0.18) between the groups. However, there were improvements in disability as assessed using the Headache Disability Index (HDI) and Neck Disability Index (NDI) scores (*p* = 0.022 and *p* = 0.001, respectively) and headache impact as assessed using HIT-6 scores (*p* = 0.037). These results favored the beneficial effect of the combination of interferential current and pharmacologic treatment to reduce disability and improve the headache impact in CTTH.

Mohamadi *et al*. [40] tested the efficacy of the positional release technique combined with ibuprofen in comparison with ibuprofen alone for 5 weeks. The frequency and intensity of headaches improved significantly in the intervention group in comparison with medical therapy alone (*p * < 0.001 for both outcomes) [40]. Another study investigated the effect of relaxation and EMG biofeedback on CTTH over 3 weeks [42]. The researchers did not apply any in the control arm. The study showed a significant improvement in headache-free days in patients who received relaxation and EMG biofeedback in comparison with the control group (*p * < 0.001). In the study by Kiran *et al*. [39] the intervention group was treated with Raj yoga and routine medical treatment for 8 weeks, and the control group received only routine medical treatment. Headache intensity, headache frequency, and headache duration improved significantly in the medical therapy combined with the Raj yoga arm compared with the medical therapy alone arm (*p * < 0.001 for all these parameters). Gopichandran *et al*. [2] applied muscle relaxation and deep breathing exercises in the intervention group for 12 weeks, and no intervention was applied in the control group. Patients with CTTH in the intervention group reported significant improvements in frequency and intensity of headache (*p * < 0.001 and *p * < 0.001, respectively) and headache impact as assessed using HIT-6 (*p * < 0.001) compared with the control group [2]. Martín-Vera *et al*. [38] performed neck-shoulder strengthening exercises on the intervention group for 12 weeks, and their control group was asked to continue their daily activities with no exercise applied. Patients with CTTH in the intervention group reported significant improvements in the duration and intensity of headaches (*p* = 0.007 and *p* = 0.001, respectively) compared with the control group.

### 3.2 Methodologic quality assessment of the studies

The methodologic quality of the studies was assessed using PEDro (Table 2, Ref. [2, 36, 37, 38, 39, 40, 41, 42, 43]). The methodologic qualities were excellent in the studies of Chassot *et al*. [42], Wang *et al*. [36], and Pérez-Llanes *et al*. [43] (scores are 10, 10 and 9 respectively). The studies by Gopichandran* et al.* [2], Martín-Vera *et al.* [38], and Mohamadi *et al*. [40] had good quality (scores are 8, 8 and 7, respectively). Only the study of Kiran *et al*. [39] had a fair quality score of 5.

**Table 2.** Methodologic quality of the trials.

	PEDro Scale Items											
Author, year	1	2	3	4	5	6	7	8	9	10	11	Total Score
Kiran *et al*. [39] 2014	YES	YES	NO	NO	YES	NO	NO	YES	NO	NO	YES	5
Rokicki *et al*. [37] 1997	YES	YES	NO	YES	NO	NO	NO	YES	NO	YES	YES	6
Mohamadi *et al*. [40] 2020	YES	YES	YES	NO	NO	NO	NO	YES	YES	YES	YES	7
Gopichandran *et al*. [2] 2021	YES	YES	YES	YES	NO	NO	NO	YES	YES	YES	YES	8
Martín-Vera *et al*. [38] 2023	YES	YES	YES	YES	NO	NO	YES	YES	NO	YES	YES	8
Berggreen *et al*. [41] 2012	YES	YES	YES	YES	YES	NO	NO	YES	YES	YES	YES	9
Pérez-Llanes *et al*. [43] 2022	YES	YES	YES	YES	YES	NO	NO	YES	YES	YES	YES	9
Wang *et al*. [36] 2007	YES	YES	YES	YES	YES	YES	YES	YES	NO	YES	YES	10
Chassot *et al*. [42] 2015	YES	YES	YES	YES	YES	NO	YES	YES	YES	YES	YES	10

*“YES” indicates the presence of the item. “NO” indicates the absence of the item. 1: Eligibility criteria were specified. 2: Subjects were randomly allocated to groups (in a crossover study, subjects were randomly allocated an order were received). 3: Allocation was concealed. 4: The groups were similar at baseline regarding the most important prognostic indicators. 5: There was blinding of all subjects. 6: There was blinding of all therapists who administered the therapy. 7: There was blinding of all assessors who measured at least one key outcome. 8: Measures of at least one key outcome were obtained from more than 85% of the subjects initially allocated. 9: All subjects for whom outcome measures were available received the treatment or control condition as allocated or, where this was not the case, data for at least one key outcome was analyzed by “intention to treat”. 10: The results of between-group statistical comparisons are reported for at least one key outcome. 11: The study provides both point measures and measures of variability for at least one key outcome. PEDro: Physiotherapy Evidence Database.*

We also assessed the methodologic qualities of studies that were not included in the meta-analysis. In the study of Berggreen *et al*. [41] the PEDro score was excellent (score is 9). The reason why this study was not included in the intensity of headache analysis was that headache was questioned in the morning and evening; therefore, it had two headache intensity parameters. In the study of Rokicki *et al*. [37] the PEDro score was good (score is 6). This study was not included in the intensity of headache analysis because it had a 0–40 scoring range. Additionally, headache frequency was evaluated as headache-free days/week. Accordingly, the methods evaluated in these studies were different from those included in the analysis.

### 3.3 Data synthesis and analysis

In this study, meta-analyses were performed using the data of the following common outcome measures from seven studies: intensity of headache in 380 patients, headache frequency in 235 patients, duration of headache in 106 patients, and headache impact in 194 patients.

#### 3.3.1 Intensity of headache

Seven studies were eligible for the quantitative analysis of the intensity of headache as assessed using VAS or NPRS [36, 38, 39, 42, 43], Numerical Pain Index (NPI) [40] or WBFPS (scores ranging from 0 to 10) [2]. The random-effects model analysis showed a significant reduction in the standardized mean difference (SMD) for headache intensity in patients who underwent physiotherapy compared to those who did not (SMD: −1.17, 95% CI (confidence interval): [−1.86 to −0.49]; *p * < 0.01) (Fig. 4, Ref. [2, 36, 38, 39, 40, 42, 43]). The low level of evidence indicates that our confidence in the effect estimate is limited (Table 3).

**Fig. 4.** Forest plot analysis showing decreases in the intensity of headache from baseline. SD: standard deviation; CI: confidence interval.

**Table 3.** Data synthesis and analysis outcomes with GRADE results.

Outcomes	Number of analyzed studies and sample size	SMD [95% CI]	Risk of Bias^a^	Inconsistency^b^	Indirectness	Imprecision^c^	Publication Bias^d^	Quality of Evidence (GRADE)
Headache Intensity	7, 380	−1.17 [−1.86; −0.49]	Serious	Serious	Not Serious	Serious	No	Low
Headache Frequency	3, 235	−1.36 [−2.47; −0.26]	Serious	Serious	Not Serious	Serious	No	Very Low
Headache Duration	3, 126	−0.71 [−1.31; −0.12	Serious	Serious	Not Serious	Serious	No	Very Low
Headache Impact	2, 195	−0.90 [−2.36; 0.56]	Serious	Serious	Not Serious	Serious	Serious	Very Low

*^a^More than 75% of the analyzed studies are not rated as low risk, ^b^I^2^ > 50%, ^c^Population < 400, ^d^Funnel plot has major asymmetry. SMD: standardized mean difference; CI: Confidence interval; GRADE: Grading of Recommendations, Assessment, Development and Evaluations.*

#### 3.3.2 Headache frequency

The studies conducted by Gopichandran *et al*. [2], Mohamadi *et al.* [40], and Martín-Vera *et al*. [38] reported headache frequency on monthly headache days and therefore data from these studies were eligible for quantitative analysis. The random-effect model analysis showed a significant reduction in headache days in patients who received muscle relaxation and did exercises with an average of 1.36 fewer headache days per month (95% CI: [−2.47 to −0.26]; *p* = 0.02) compared with those who did not (Fig. 5, Ref. [2, 38, 40]). The very low level of evidence indicates that our confidence in the effect estimate is very limited (Table 3).

**Fig. 5.** Forest plot analysis showing decreases in the frequency of monthly headache days from baseline. SD: standard deviation; CI: confidence interval.

#### 3.3.3 Headache duration

Three studies [36, 38, 39] evaluating daily hours of headache were eligible for quantitative analysis of headache durations. The random-effect model analysis showed the reduction in SMD of the headache duration was significantly greater in patients who received Raj yoga or acupuncture or neck-shoulder strengthen exercise compared with those who did not (SMD: −0.71, 95% CI: [−1.31 to −0.12]; *p* = 0.02) (Fig. 6, Ref. [36, 38, 39]). The very low level of evidence indicates that our confidence in the effect estimate is very limited (Table 3).

**Fig. 6.** Forest plot analysis showing decreases from baseline in the duration of the headache. SD: standard deviation; CI: confidence interval.

#### 3.3.4 Headache impact

Three studies were eligible for analysis of headache impact using the HIT-6 questionnaire [2, 43]. The random-effect model analysis of the data favored the improvement in headache impact in patients who received muscle relaxation compared with those who did not. However, the decrease in SMD values of HIT-6 scores in each arm did not reach statistical significance (SMD: −0.90, 95% CI: [−2.36 to 0.56]; *p* = 0.23) (Fig. 7, Ref. [2, 43]). The very low level of evidence indicates that our confidence in the effect estimate is very limited (Table 3).

**Fig. 7.** Forest plot analysis showing decreases from baseline in the HIT-6 scores. SD: standard deviation; CI: confidence interval.

## 4. Discussion

This study systematically reviewed nine RCTs that met the eligibility criteria concerning the effectiveness of physiotherapy approaches in CTTH. Seven studies on headache intensity, three studies on headache frequency, three studies on duration, and two studies on headache impact were included in the meta-analysis. In the results, headache intensity, frequency and duration showed significant improvement after physiotherapy approaches.

Nine RCTs included intervention arms featuring techniques such as neck-shoulder strengthen exercises, acupuncture-like electrical stimulation, trigger point massage, suboccipital muscle inhibition combined with interferential current, electroacupuncture, positional release technique, relaxation and EMG biofeedback, Raj Yoga, muscle relaxation, and deep breathing exercises. Control arms comprised sham acupuncture-like electrical stimulation, analgesics, and muscle relaxants in some of these studies, or no treatment or intervention was applied. Overall, the included articles demonstrated positive results indicating improvement in evaluated parameters compared with control arms. In general, acupuncture, muscle relaxation methods (including Raj yoga, suboccipital relaxation, and interference current), and neck-shoulder exercises provided significant improvement in headache intensity. However, according to GRADE, headache intensity had a low level of evidence and our confidence in the effect estimate is limited. Muscle relaxation methods and neck-shoulder exercises provided significant improvement in headache frequency, and muscle relaxation methods (including Raj yoga) and neck-shoulder exercises provided significant improvement in headache duration. However, for these two parameters, the evidence levels were very low according to GRADE. The actual effect may differ greatly from the effect estimate. To further address the magnitude of treatment effects, the standardized mean differences (SMD) for key outcomes such as headache intensity, frequency, and duration showed statistically significant improvements. For headache intensity, the SMD was −1.17 (95% CI: −1.86 to −0.49), indicating a large treatment effect, though the wide confidence interval suggests some variability, likely due to differences in study designs and small sample sizes. Similarly, headache frequency showed a reduction of 1.36 headache days per month (95% CI: −2.47 to −0.26), again reflecting a positive result, but with notable variability. These wide confidence intervals and the small sample sizes in the studies raise concerns about the stability and reliability of the observed effects. Our GRADE assessments confirm that the evidence for most outcomes is of low or very low quality, further indicating the need for cautious interpretation. The findings, while promising, highlight the importance of larger-scale, well-designed studies to validate the observed effects and improve the confidence in these estimates.

When the literature is examined, the decreases in the intensity of headaches are greater in intervention groups than in control groups [2, 36, 38, 39, 40, 42, 43]. Treatments in the included studies included approaches targeting relaxation of the suboccipital muscles and neck muscles. Although TTH is defined as a headache that wraps the head like a band, cranio-cervical muscle sensitization is one of the common findings in CTTH. Sollman* et al*. [44] detected increased neurogenic inflammation in the trapezius muscle, which is one of the most common myofascial symptoms in patients with muscle tension headaches, using magnetic resonance imaging with T2 mapping, and stated that approaches to peripheral sensitivity in myofascial tissues could be targeted. The favorable effect of physiotherapy interventions on the intensity of headaches was confirmed by the quantitative analysis of the available data [2, 36, 38, 39, 40, 42, 43]. Therefore, it may serve as a warning that we should target the muscles throughout the head, starting from the neck, in approaches aimed at reducing the intensity of pain in TTHs. It is stated that the peripheral sensitivity of nociceptors in the craniocervical muscles and the sensitivity developed by long-term stimulation of nociceptive pain pathways in the central nervous system may be one of the factors that causes the chronification of TTHs [45]. Considering the anatomic, neurologic, and, functional relationship of the head and neck region, the tension of the muscle structures may be reflected in each other, and muscle relaxation approaches that include the neck may provide positive benefits on the intensity of headache in patients with CTTH, who are more likely to recognize nociceptive stimuli and have more experience of experiencing pain. They have poor pain control, requiring higher stimulation to reach the onset and peak amplitude of the electrophysiological sensory nerve action potential. Therefore, the fact that physiotherapy approaches target pain and achieves successful results suggests that physiotherapy approaches are promising in the treatment of CTTH and should be supported by electrophysiologic approaches in future research [46].

Considering studies evaluating headache frequency, it was reported that the decreases were greater in the intervention groups than in the control groups. Although heterogeneity results in a low-grade level of evidence, quantitative analysis of the data from studies evaluating the frequency of headaches revealed that muscle relaxation practices significantly reduced the number of headache days per month [2, 40]. Physiotherapy approaches encompass a diverse range of techniques, including stabilization, stretching, relaxation exercises, and aerobic exercises for the head and neck. Implementing these approaches over a period exceeding 12 weeks, rather than a shorter 4-week duration, may potentially enhance the effect on headache frequency. Prolonged exercise interventions, such as 12 weeks, have been shown to increase well-being in patients with CTTH [47]. Quantitative analysis of two studies focusing on headache duration in hours per day indicated neck-shoulder strength exercises [38], acupuncture [36], and muscle relaxation [39] approaches had a significant impact on headache duration in the intervention groups compared with the control groups. Although the articles contain heterogeneous approaches, the significant reduction in headache duration is promising. Positive results can be obtained by identifying missing points in future studies and conducting quality randomized controlled studies to eliminate this heterogeneity. Given the importance of muscle sensitivity in CTTH, the application of touch-based physiotherapy approaches that affect tissue may offer recovery benefits that warrant further investigation.

CTTH is characterized by experiencing headaches for more than 15 days per month, lasting from hours to days [1]. Studies indicate that a low-pressure pain threshold in craniocervical muscles, resulting from pain pathway sensitivity, correlates with headache intensity and frequency in patients with TTH [7, 48]. The association of central sensitization with chronic pain underscores the importance of long-term and follow-up treatments in addressing prolonged patient symptoms. In a study by Castien *et al*. [47], where 8-week cervical-thoracic exercises, postural regulation exercises, and mobilizations were performed on patients with CTTH, there was a significant decrease in headache frequency, duration, and intensity. Thus, it is advisable to explore the effectiveness of long-term physiotherapy programs aimed at reducing monthly headache duration.

Muscle relaxation approaches [2, 43] applied in the evaluation of headache impact did not yield statistically significant results in the meta-analysis. Despite significant improvements in the headache impact within both intervention groups, variations in the applied muscle relaxation techniques, treatment durations, sample sizes, and limited common outcome measures may explain the lack of significance in the result. The headache impact may involve several parameters that affect the quality of life in daily life. These include physical, functional, social, and emotional aspects, as well as emotional well-being, energy/fatigue in daily tasks, concentration, pain and general health perception [49, 50]. Patients with CTTH also have negative effects on daily tasks [51]. Consequently, the multifaceted nature of headache impact and quality of life may require extended treatment durations to effectively address monthly headache frequency and quality of life in CTTHs. Thus, careful consideration and structuring of treatment approaches are essential to investigate the healing effectiveness of the targeted parameters in CTTHs.

In this systematic review and meta-analysis on CTTH, the studies were inadequate in terms of sufficient sample size, duration of treatment, and applications. A sample size of less than 400 detected in outcome measures at the GRADE evidence level is seen as a serious issue that needs to be addressed. Therefore, this situation is also reflected in the analysis results. It can be understood from the results that larger sample sizes will better represent the population.

It appears that acupuncture is effective in reducing the intensity of headaches, headache duration and headache frequency [36, 42]. Various stimuli, whether harmful or harmless, may lead to sensitization and muscle tenderness by activating A-delta and C fibers in TTH [52]. Continuous stimulation can trigger chronic conditions by activating the central nervous system. Increased nociceptor activity in the pericranial muscles causes muscle tenderness and stiffness, resulting in hypersensitive trigger points in the muscles [5]. Acupuncture also increases the stimulation threshold of nociceptive neurons by stimulating these sensitive muscles [36]. The circulation of the tissue increases and the removal of chemicals that increase excitability in the tissue can reduce pain [36]. Trigger point treatment, on the other hand, creates local and temporary hypoxia in the tissue, aiming to reduce vascular spasms and regulate circulation, preventing the activation of sensitive points [53]. Muscle relaxation methods use mechanical stimulation to activate the gate control mechanism of pain, increase beta-endorphin levels, raise the pain threshold, and decrease the sensation of pain. Mechanoreceptors are activated, facilitating muscle relaxation by increasing tissue flexibility and reducing tension and spasms [53, 54, 55]. The observed efficacy of physiotherapy in patients with CTTH is attributed to their effects of enhancing the nociceptor activity, mechanoreceptors, and tissue circulation over short periods [36, 53, 54, 55, 56]. It seems that pericranial muscle tenderness and lower pressure pain level, which is a characteristic feature in CTTH, can be improved by these mechanisms of action [7]. The fact that deep cervical muscle thickness, craniovertebral angle, and cervical joint range of motion are less in patients with TTHs compared with healthy controls reveals that exercise approaches targeting the spine should be regularly applied in treatments for the management of the disease process [8, 11, 12, 13]. Inhibition of deep neck muscles as a result of decreased alpha motor neuron activity may increase the load on superficial neck muscles and cause a change in activation. Therefore, cervical stabilization is impaired. With exercises, alpha motor neuron activation is increased in deep cervical muscles, muscle tone is reduced, joints adaptively change direction, muscles are strengthened, and the cervical spine is reshaped. As a result, the aim is to achieve cervical stabilization [57].

None of the studies reported adverse effects. It is noteworthy that physiotherapy approaches, when used in conjunction with medical treatment, offer support to patients with few adverse effects, keeping them active and safe. Given the chronic nature of the problem, the authors emphasize the importance of using safe physiotherapy approaches in patients with CTTH to prevent potential future issues, such as overreliance on medication [2]. In addition, the combined application of exercise programs and medical treatments may provide treatment response and improvement in symptoms. In patients with migraine, an increase in physical activity based on daily step count is positively associated with the treatment response to monoclonal antibodies. This information is also valid for CTTHs [58].

### 4.1 Limitations

In this study, nine RCTs met the inclusion criteria for the systematic review. However, not all of these studies could not be included in the analysis due to differences in the methods used and few common outcome measures.

In our previous systematic review and meta-analysis [29] we found that physiotherapy approaches were effective in reducing headache intensity and headache days per month in patients with chronic migraine. However, the studies had limitations such as heterogeneity in treatment content and duration, as well as insufficient sample sizes [29]. In the current research, the studies face similar limitations, in terms of sample size, duration of treatment, and approach consistency.

It was observed that the physiotherapy approaches used were mainly electroacupuncture, muscle relaxation and exercise methods. However, the articles generally differ in terms of sample sizes, interventions applied, treatment durations, and outcome measures. Therefore, we had difficulty grouping the studies and interpreting their analysis due to the lack of common content. This is another limitation of our study.

Although the evaluation parameters in the studies included baseline results of parameters such as quality of life and disability, the results at the end of the treatment were not given, which caused these parameters not to be included in the analyses. Therefore, the number of articles was limited in evaluating the parameters.

There was no common period in the studies in terms of follow-up period, and although some studies did not give a follow-up period, some gave short follow-up periods and some had long follow-up periods. Therefore, because there was no common time interval, we were not able to conduct an analysis in which we could evaluate the follow-up period.

### 4.2 Implications

Suggest future directions: The limitations identified in our research underscore the necessity for more research in this domain. Future studies should endeavor to conduct RCTs employing diverse and comprehensive physiotherapy approaches, including exercises and manual therapies, with larger sample sizes and longer follow-up periods, along with a comprehensive evaluation of relevant parameters.

Physiotherapy methods as adjunctive treatment: Our study’s results highlight the benefits of physiotherapy approaches in ameliorating headache symptoms in patients with CTTH, either in addition to pharmacologic treatment or as adjunctive treatment when pharmacologic approaches are insufficient or intolerable. A combined approach of pharmacologic and non-pharmacologic interventions could ensure a more holistic and effective treatment process for both patients with CTTH and healthcare professionals.

Multidisciplinary approach: While acknowledging the limited scope of physiotherapy studies in CTTH, we emphasize the significance of a multidisciplinary approach in the evaluation and treatment of patients with CTTH. Quality randomized controlled treatment studies conducted collaboratively by healthcare professionals in neurology and physiotherapy are essential to bridge existing gaps [59]. The synergy of knowledge from these two domains could result in innovative treatment protocols, emphasizing the importance of collaboration.

Adverse effects: Although adverse effects were not reported in the reviewed studies, addressing possible adverse effects and precautions is crucial for future studies considering these interventions.

ICHD diagnosis criteria: The review highlighted that only one study did not address the diagnostic criteria for ICHD [2], and four studies did not specify the ICHD edition they referred to [36, 39, 40]. To ensure standardization in patient evaluation, we recommend that future studies meticulously apply and detail the ICHD diagnostic criteria in their presentations.

## 5. Conclusions

In summary, based on the review of articles meeting inclusion criteria and the results of the meta-analysis, exercises, electroacupuncture, and muscle relaxation methods within physiotherapy approaches are effective in reducing the intensity, frequency, and duration of headaches in CTTH. This aligns with expectations given the muscle tenderness often associated with CTTH.

## Supplementary Material

Supplementary material associated with this article can be found, in the online version, at https://files.jofph.com/ files/article/1899697906390056960/attachment/ Supplementary%20material.docx.


